# Patient’s safety culture among health care professionals in Tunisian Center-Est University Hospital

**DOI:** 10.1186/2047-2994-4-S1-P271

**Published:** 2015-06-16

**Authors:** N Bouafia, M Mahjoub, R Hellali, W Bannour, A Bencheikh, O Ezzi, A Asma, Z Nawel, N Mansor

**Affiliations:** 1 Hospital Hygiene, University Hospital Farhat Hached Sousse Tunisia, Jawhara, Tunisia

## Introduction

Nowadays, patients’ security arouses more and more decision-makers and health workers. Development of safety culture is fundamental pillar to any strategy for improving quality and safety care. Thus, we conducted our work in order to measure level of patients’ safety culture among healthcare professionals, in our hospital

## Objectives

Measure level of patients’ safety culture among healthcare professionals in order to improve strategies of healthcare quality and safety in our hospital.

## Methods

We conducted, in 2013, a descriptive study among all licensed physicians (n= 116) and a representative sample of paramedical staff (n= 203) exercising at university hospital center Farhat Hached Sousse (Tunisia). Measuring instrument used is a valid questionnaire containing ten safety care dimensions.

## Results

Participation rate was 90.5%.

44.9 % of respondents felt that security level of their services is low. Overall score of different dimensions varies between 32.7% and 68.8%. Dimension having most developed score (68.8%) was perception of “Frequency and reporting adverse events”. Dimension with lowest score (32.7 %) was “Management support for safety care”.

## Conclusion

Our study has allowed us to conclude that all dimensions of patients’ safety culture need to be improved among our establishment’s professionals. Therefore, more efforts are necessary in order to develop a security culture based on confidence, learning, communication and team work and rejecting sanction, blame, criminalization and punitive reporting.

## Disclosure of interest

None declared.

